# Making birthing safe for Pakistan women: a cluster randomized trial

**DOI:** 10.1186/1471-2393-12-67

**Published:** 2012-07-15

**Authors:** Muhammad Amir Khan, Shirin Mirza, Maqsood Ahmed, Akhtar Rasheed, Amanullah Khan, John Walley, Nailah Nisar

**Affiliations:** 1 Association for Social Development, Islamabad, Pakistan; 2 Provincial Programme for Family planning and Primary health care (Lady Health workers programme) for Punjab, Lahore, Pakistan; 3 Save the Children, Islamabad, Pakistan; 4 Nuffield Centre for International Health and Development, Leeds Institute of Health Sciences, University of Leeds, Leeds, UK; 5 The Royal Surrey County Hospital NHS Foundation Trust, Surrey, UK

## Abstract

**Background:**

Two out of three neonatal deaths occur in just 10 countries and Pakistan stands third among them. Maternal mortality is also high with most deaths occurring during labor, birth, and first few hours after birth. Enhanced access and utilization of skilled delivery and emergency obstetric care is the demonstrated strategy in reducing maternal and neonatal mortality. This trial aims to compare reduction in neonate mortality and utilization of available safe birthing and Emergency Obstetric and Neonatal Care services among pregnant mothers receiving ‘structured birth planning’, and/or ‘transport facilitation’ compared to routine care.

**Methods:**

A pragmatic cluster randomized trial, with qualitative and economic studies, will be conducted in Jhang, Chiniot and Khanewal districts of Punjab, Pakistan, from February 2011 to May 2013. At least 29,295 pregnancies will be registered in the three arms, seven clusters per arm; 1) structured birth planning and travel facilitation, 2) structured birth planning, and 3) control arm. Trial will be conducted through the Lady Health Worker program. Main outcomes are difference in neonatal mortality and service utilization; maternal mortality being the secondary outcome. Cluster level analysis will be done according to intention-to-treat.

**Discussion:**

A nationwide network of about 100,000 lady health workers is already involved in antenatal and postnatal care of pregnant women. They also act as “gatekeepers” for the child birthing services. This gate keeping role mainly includes counseling and referral for skill birth attendance and travel arrangements for emergency obstetric care (if required). The review of current arrangements and practices show that the care delivery process needs enhancement to include adequate information provision as well as informed “decision” making and planned “action” by the pregnant women. The proposed three-year research is to develop, through national technical working group process, and then test a set of arrangements for achieving the enhanced utilization of safe birthing services.

**Trial registration:**

Current Controlled Trials ISRCTN86264432

## Background

### Burden of Neonatal and maternal mortality in Pakistan

Around four out of 30 million infants born each year worldwide die in the first 28 days of life and more than one quarter of deaths occur in the first 24 hours of birth [1,2]. Two out of three neonatal deaths occur in just 10 countries and Pakistan stands third among these countries [1]. In Pakistan the newborn mortality is 55 deaths per 1000 live births, infant mortality rate is 77 deaths per 1000 live births and maternal mortality ratio is 276 per 100,000 live births [3]. The new born babies and infants die mainly due to birth asphyxia, intra-uterine growth retardation, and acute infections [3-5]. The major causes of maternal deaths are hemorrhage, obstructed labor, puerperal sepsis and toxemia of pregnancy. Most of these causes are both preventable and treatable.

### Continuum of care during pregnancy

Throughout the continuum of care, the period with the highest risk of death and disability for both mother and newborn is labor, birth, and the first few hours after birth. Hence the health and survival of mother and newborn are closely linked to care the mother receives before and during childbirth and during the immediate postnatal period. There is an inverse relationship between the proportion of deliveries assisted by a skilled birth attendant and the maternal mortality ratio in developing countries [6]. Enhanced access and utilization of skilled delivery care and emergency obstetric and neonatal care (EmONC) is the demonstrated strategy in countries that have succeeded in reducing maternal and neonatal mortality [7].

Studies on maternal mortality have identified the need for addressing the three delays in accessing emergency obstetric care and for integrated efforts for saving mothers’ lives both at the community and hospital level [8]. The past investments in expanded health care infrastructure have brought the services closer to the people but still majority of births occur at home and are attended by untrained traditional birth attendants [3].

### Maternal, Newborn and Child Health (MNCH) and Lady Health Worker (LHW) program in Pakistan

The Government of Pakistan has launched the Maternal, Newborn and Child Health (MNCH) Programme to address service availability and the utilization gaps, by building on the already existing experiences, initiatives and resources. The main objectives of the MNCH Programme, to be achieved by 2015, include: reducing the newborn mortality to less than 40 per 1000 live births and maternal mortality ratio to less than 140 per 100,000 live births [9]. The MNCH Programme, in accordance with the national policy, emphasizes prenatal, postnatal and newborn care, birth preparedness, skilled birth attendance, and strengthening the primary and secondary level of health care [9]. Making birth practices safer for mother and newborn is the key programme strategy for achieving the desired reduction in the maternal and newborn mortality. The Lady Health Worker (LHW) Programme evaluation has shown a reasonable coverage and uptake of LHW antenatal services in their respective communities. National Maternal and Child Health Policy and Strategic Framework (2005–2015) identifies LHWs as the core actor in addressing decision and access delays to safe child birthing [9]. So in addition to providing antenatal and postnatal services to pregnant women, LHWs also act as a ‘gate-keeper’ for safe birthing services. The LHWs carry out this gate keeping role through birth-preparedness planning (BPP) and complication-readiness planning (CRP) with pregnant women and their family members [10,11]. Currently, LHWs lack structured guidelines and tools to carry out counseling, planning and facilitation for the gate-keeping role.

### Rationale and Objectives

The proposed research exercise will focus on innovative operations and materials to reduce neonatal mortality and optimize the utilization of safe birthing services and the basic and comprehensive EmONC services being made available at the rural health centers and hospitals. The two main dimensions of the intervention to be developed and tested are: a) Enabling the pregnant women and families to plan and prepare for ‘safe birth’ and ‘EmONC’ (i.e. address the delay in decision-making); b) Mobilizing the communities to arrange travel for emergency obstetric care (i.e. address the delay in timely access).

The experience and the products of this research will help to strengthen the linkages between the LHW and MNCH programmes, in a holistic way and to build the district and programme capacity to implement these interventions under routine programme circumstances.

#### Specific objectives

 · To compare the neonatal mortality in the intervention arms as compared to the control arm.

 · To compare the utilization of safe birthing services and the basic and comprehensive EmONC services in the intervention arms as compared to the control arm.

 · To compare the maternal mortality in the intervention arms as compared to the control arm.

 · To compare the health services and the patient costs of the three selected strategies in achieving the desirable service utilization and pregnancy outcomes, and to assess the feasibility of addressing the “decision” and “access” delays in the context of the national programme in Pakistan.

 · To understand the attitudes, the social pressures (subjective norms) and the enabling factors (environment: services access and quality, time, money etc.,) that influenced the decisions and ability of pregnant women and their families to utilize the available safe birthing and EmONC services, and to suggest further improvements for the utilization of MNCH services in Pakistan.

## Methods

### Study Design

**Figure 1:**
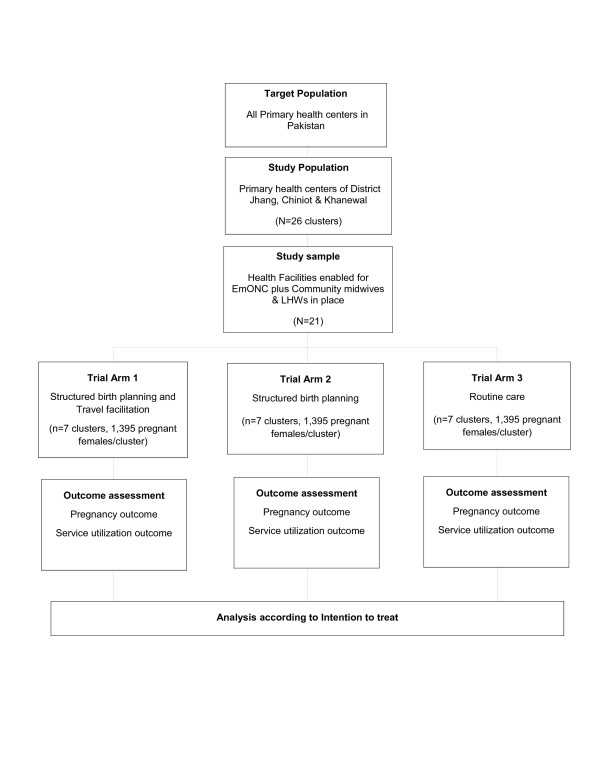
Flow of the trial.

A pragmatic parallel arms cluster randomized controlled trial (c RCT) with three arms will be conducted from February 2011 to May 2013. Pregnant females in arm 1 will be given interventions to address decision delay plus the access delay. Those in trial arm 2 will be given intervention to address decision delay only. Trial arm 3 will be the control arm (routine care of the pregnant females by the LHWs) (Figure 1).

### Interventions

The ‘structured birth planning’ intervention is to address the decision delays for safe birthing, whereas ‘transport facilitation’ intervention is to address the access delays for EmONC services.

#### Structured Birth Planning

This event, with the help of a specially designed communication guide/tool, will provide the pregnant women and family members with the required knowledge (technical, administrative and costs) and facilitate them to take informed decisions regarding safe birthing and EmONC services as required. This communication will provide the client information regarding all the available and qualified birthing service providers, in the order of programme guided preference (i.e. facility-based doctors and Lady Health Visitor (LHV), community-based Community Mid Wife (CMW), and private clinics). The agreed choice will be recorded, and pregnant women will be provided with an information package relevant to her decisions and subsequent actions.

#### Transport facilitation

The proposed intervention is a facilitated travel arrangement for pregnant woman who need to access EmONC services at the designated facility. The intervention is to ensure that the affordable (even subsidized or free for the deserving/poor women) transport facility is available in-time for the pregnant woman to access the EmONC services, when required. The arrangements for facilitated travel for EmONC will be discussed, agreed and documented during initial interaction between the pregnant woman and her lady health worker. The detailed modalities (including transport selection, subsidy, payment, communication etc.) for managing this service will be developed in consultation with districts, facilities and communities during the first few months of the proposed project. The role of EmONC facility staff in establishing and managing the travel-facilitation will be encouraged.

#### Mapping of skilled birth attendants, EmONC facilities and transport providers

In each of the selected intervention cluster, the mapping exercise will be carried out through the respective LHWs. This mapping exercise will focus on available skilled birth attendants and EmONC facilities plus available travel services in the clusters. The access, quality and cost dimensions of these available service options will be considered for a pregnant woman and family to make an informed choice. The research intervention will also supplement (but neither duplicate nor expand) the programme EmONC facility strengthening inputs, where needed.

The data collected from the mapping exercise will be organized in form of directories (Birth providers directory, transport providers directory & EmONC facilities directory) and used in the communication with pregnant women in the respective cluster. Safe birthing planning, by pregnant woman and her family, will be carried out by the LHW with each identified pregnancy in her respective locality by specially designed pictorial tool. Each woman in a cluster will be offered the same care package. As LHWs are the source to deliver the trial intervention, the chances of inter-cluster contamination are considered reasonably low.

### Outcome indicators

Two main outcome indicators to be used in the trial are pregnancy outcomes and service utilization:

***Pregnancy outcome*** is the proportion of pregnancies that results in neonatal (and maternal) deaths.

***Service utilization indicators*** are mainly the proportion of pregnancies that deliver by skilled birth attendants and those who access the EmONC facilities.

The comparison of these indicators between the intervention and control arms will determine the effectiveness of the interventions in terms of enhanced service utilization and reduced mortality.

### Study Setting

Two districts of Punjab i.e. Jhang and Khanewal have been selected for implementing the proposed trial. These are the districts where MNCH Programme or its partners have carried out the facility strengthening for EmONC services, have trained two or more batches of community midwives, and have a functioning network of lady health workers.

 · *Jhang* (recently divided into Jhang and Chiniot) is spread over an area of 8809 square kilometers, with an estimated population of about 3.5 million, and male to female ratio of 1.08. One quarter of the population is urban, whereas three quarters is rural. Total literacy rate is 37.12%, with 51.53% in males and 21.43% in females. The average household size is 6.5 with 85.9% dependency ratio. Currently married population is 62.2%.

 · *Khanewal* is spread over an area of 43494 square kilometers, with an estimated population of about 2,503,459 with 52% males and 48% females. The estimated population growth rate is 2.4%. The infant mortality rate is estimated to be 86/1000 which is higher than the provincial as well as the national figure of 77/1000. Total literacy rate is 45% and 33% of the population is living below poverty line. The crude birth rate is 21.5 and average household size is 6.9. It has four tehsils Khanewal, Mian Channu, Kabirwala and Jahanian.

### Study Population

In these two districts, there are about 26 EmONC facilities (tehsil head quarter hospitals (THQ) and rural health centers (RHC)), out of which *21* will be selected into the trial mainly on the basis of: a) general health care and EmONC service assessment, and b) both facility and community consent to participate in the trial. Each selected THQ and RHC, along with its associated BHUs and catchment population, will form a cluster for the trial. All pregnant women in these catchment populations (regardless of their age, social or medical profile) will be offered the services, as per the trial protocol.

### Data Management procedures

Most of the data will be collected as a part of the care delivery process by LHWs on the ‘Mother and Child Health Card’. The structured planning of safe birthing will be documented on specially designed ‘Birth Plan Form’. A copy of each of these birth plan data will be submitted to the respective health facility on a monthly basis, during the routine visit of the respective LHW. Each form will be reviewed for completeness, and those found with missing data will be acted upon accordingly. A sample of the filled/submitted forms will be cross-checked by the lady health supervisor (LHS), during her routine visit to the respective community. Similarly the transport facilitation plan will also be documented and records will be maintained.

EmONC service utilization and outcome data will be extracted from the relevant EmONC records being kept at the facilities. The EmONC data extracted from the facility record will also be cross-matched with data to be received from the respective LHWs. Research assistants will collect the data on monthly basis from the selected facilities in all the trail arms and send it to the central trial unit for data entry, cleaning and analysis.

Verbal autopsy will be conducted for 10% of the reported neonatal (and maternal) deaths, to understand their care experiences and the circumstances leading to death. A trained project staff, facilitated by the respective LHW, will carry out the verbal autopsy, within a month of the death being reported by the LHW. A specially designed tool will be used.

### Statistical considerations

The estimated required sample size for the trial is twenty one clusters. A strengthened EmONC facility (i.e. THQ and RHC) along with associated BHUs and catchment population (approximately 170,000) is taken as a ‘cluster’ for the trial. The required sample size has been estimated in light of internationally recommended procedure for field trial of health interventions [12]. This sample size gives 80% power to detect the assumed outcome differences (i.e. reduction in neonatal mortality by 25%) between the control and intervention arms. The acceptable confidence level is 95% and coefficient of variation in true proportion (k) is 0.17. Taking ICC as 0.0012, and loss to follow-up as 20%, at least 1,395 pregnant females will be required per cluster, at least 9,765 pregnant females per arm; a total of at least 29,295 pregnant females in the three trial arms.

The results in each cluster will be analyzed on the basis of “intention to treat”. This means the service utilization and pregnancy outcomes of each woman will be counted in the intervention for the cluster, regardless of her actually accepting or availing the offered services.

### Costing Study

Two main types of costs (i.e. health services and consumer) will be combined to get the intervention costs for the three options being assessed in the trial. The ‘increased service utilization’ and ‘reduced neonatal and maternal mortality’ will be used as the main measure of effectiveness, and cost effectiveness is defined in terms of cost per additional birth by a skilled attendant and cost per neonatal and maternal death averted.

### Exploratory Qualitative Study

The study will collect data on a range of social, economic, service access and quality etc. factors from care providers as well as pregnant women and their families to assess the social and administrative feasibility of said interventions in the programme context and inform refinements before scaling-up to other parts of the country.

### Addressing bias, error and limitations

Comparing the socio-demographic and health services profiles of the three selected districts does not indicate any significant difference that can potentially lead to differences in care seeking behavior and/or quality of service. The proposed cluster randomization is expected to address the potential selection bias across the three trial arms.

The lack of masking (blinding) could introduce bias through two routes i.e. “incorrect measurement” and “leakage” from one group to another. There is a possibility of incorrect measurement of “service utilization” and “pregnancy outcomes” by the lady health worker, who is aware of the intervention allocation. This possibility of measurement bias will be reduced through better training and vigilant supervision of lady health workers as well as cross-verifying a sample of these measurements by the respective lady health supervisor (also research staff, where possible). Although this is possible, we believe this is unlikely that pregnant woman in the control arm would somehow get “structured birth planning” and/or “travel facilitation” from lady health worker in an intervention arm (i.e. LHW associated with another facility and serving some other population) leading to misinterpretation of results.

The possible poor quality of EmONC services available at the facilities could lead to an under-estimation of the potential gain from the trial interventions to address the decision and access delays. Before starting the trial, efforts will be made, mainly through programme but supplemented with minimal project inputs (if required), to make acceptable quality EmONC services available at the *twenty one* selected facilities. This facility support (if made available) will be same for all the facilities, thus not introducing any bias in the measurement of association. The cluster randomization will also ensure an acceptable distribution of any possible effect, of varied service quality, across three trial arms and thus avoiding getting any false association.

The study size is adequate for valid measurement of differences in service utilization and neonatal mortality. However, to get valid measurement of differences in maternal mortality the required sample size is 66 (instead of 7) clusters per arm or 198 total in the trial (instead of 21). This more than ten-fold increase in sample size is not feasible to manage, so neonatal mortality is taken as the key pregnancy outcome indicator.

The proposed randomization does not allow asking and responding to individual preferences/convenience. So the trial will not answer the question what would happen if health services are able to offer intervention according to patient preferences/convenience. Further work will be needed to test the approaches sensitive to individual preferences, in the local programme setting.

### Ethical Considerations

The trial will be conducted after taking communal consent from the trial clusters. To get communal consent, the district health office will organize an event, at each selected EmONC facility. The proposed key leaders (types) to be involved in the communal consent process/event may include: headmistress of the local girls school, local body representatives (male and female), and two notables from the local communities (i.e. one man and one woman), and one or two civil society representatives. Even after communal consent, each pregnant woman in trial clusters will be asked for permission to plan the birthing and EmONC services. In case of refusal, their data is recorded along with the reason for refusal (where possible).

National Bioethics Committee (NBC) Pakistan has granted ethical approval for the proposed project (Ref No. 4-87/10NBC-39/RDC/487). The trial has also been registered with the Current Controlled Trials ISRCTN86264432.

### Dissemination plan

The results/experiences and products of research will also be shared with wider stakeholders through: a) existing partner networks (e.g. COMDIS), b) presentations at national and international conferences (e.g. White Ribbon Alliance, World Federation of Public Health Associations), and c) access to resource centers/websites more widely used e.g. WHO.

The scientific papers, based on results of the trial and associated studies, will be submitted to international and national peer-reviewed journals. The potential journals to target includes: BMC Women Health, Lancet, British Medical Journal, WHO Bulletin, International Journal of Gynecology and Obstetrics - overall results of the trial; BMC Implementation Science - process evaluation study(s); Health Policy and Planning, Reproductive Health Matters - social science (qualitative studies)

## **Discussion**

In Pakistan, the Ministry of Health is a policy development and national level planning body. It has established a Maternal and Newborn Health (MNH) Cell to coordinate integrate and streamline all MNH activities in the country. The district health offices are responsible for implementation, monitoring and management of healthcare delivery including MNCH, through a network of 40-60 bed Tehsil Head-Quarter hospitals (THQs), 20 bed Rural Health Centers (RHCs) and 4-6 Basic Health Units (BHUs) around each RHC. The provincial departments of health, in accordance with national policies and with support of the National MNCH Programme, have started: a) training community midwives for enhanced coverage of skilled birth attendants, and b) strengthening the RHCs and THQs for basic emergency obstetric care and district headquarter hospitals for comprehensive emergency obstetric care. These birthing and EmONC services are also being provided by private facilities in each district. However, the quality and cost of services at private facilities vary widely.

Currently at the community level, the birthing services are provided by a wide range of qualified, semi-qualified, and unqualified birth attendants. The coverage of qualified skilled birth attendant is still less than the semi- or non-skilled attendants. An increased utilization of the available skilled birth attendants implies that pregnant woman is: a) informed about the safe birthing options available, b) able to select the feasible choice, and then c) able to avail the feasible safe birthing services (when required). Similarly an increased utilization of EmONC care also implies that the woman is: a) informed about what emergency obstetric care may be required: when and from where, b) able to decide when and from where to seek EmONC care (if required), and then c) able to reach in-time the right facilities for EmONC care.

The National Program for Family Planning and Primary Health Care (NP FP&PHC) has deployed a network of LHWs for delivering basic primary health care, with particular focus on reproductive health care through community outreach. Each LHW provides services to a 1000 population, and is paid a monthly stipend by the Programme. The LHWs, in catchment population of a health facility, are linked to the respective facility for regular material replenishment and technical supervision/support through the facility. In addition, each cluster of 20 – 30 lady health workers also receives field support from the respective lady health supervisor.

The LHWs are responsible for identifying and registering all pregnant women in their catchment population and encourage them to visit the nearest health facility for antenatal and postnatal care. In case the pregnant woman is unable to go to the health center every month, she is encouraged for at least 4 antenatal check ups during the pregnancy i.e. first visit during first 4 months, the second visit during 6^th^ and 7^th^ month and third and fourth visits during 9^th^ month of pregnancy. They also carry out the gate keeping role for the safe birthing services, by counseling, referring and facilitating their access to safe birthing services. However, the review of current arrangements shows that their enabling of pregnant woman is limited to information provision about service availability, with minimal work on actual “decision” and “action”. This inadequacy in gate keeping role is mainly because the lady health workers lack the guidelines, tools and communication materials to cover all three dimensions of woman enabling i.e. information, decision, and action.

The proposed research will address this woman enabling gap by developing feasible and replicable guidelines and materials for the LHWs to provide each pregnant woman: a) adequate information about safe birthing and EmONC services, b) an opportunity for her to influence the selection of safer services to avail (i.e. from where, when and how), and c) an opportunity for her to get the agreed birthing and EmONC services planned/arranged in-advance for her well-being.

## Competing interest

The authors declare that they have no competing interest (financial or otherwise) in this publication.

## Authors’ contribution

MAK carried out the background literature review, identified the research gap, conceived the research question and objectives and proposed the trial. MA and SM contributed towards study methodology, manuscript writing and its critical review for intellectual content. AR helped in designing the trial in LHW programme context and gave technical guidance for tool development in the intervention arms. AK provided technical inputs for community based care components for maternal and child health care and guided the design of travel facilitation intervention. JW reviewed the study design with regards to the health systems component. NN contributed obstetrics knowledge for the study design. All the authors approved the final manuscript.

## Pre-publication history

The pre-publication history for this paper can be accessed here:

http://www.biomedcentral.com/1471-2393/12/67/prepub
